# Evaluation of *Haemophilus influenzae* type b carrier status among children 10 years after the introduction of Hib vaccine in Brazil

**DOI:** 10.1590/0074-02760150140

**Published:** 2015-09

**Authors:** Rosemeire Cobo Zanella, Maria Cristina de Cunto Brandileone, Ana Lúcia Andrade, Cinthya Terumi Ogassavara, Cleiton Eduardo Fiório, Angela Pires Brandão, Samanta Cristine Grassi Almeida, Ana Paula Silva Lemos, Maria Cecília Gorla, Telma Regina Carvalhanas, Helena Sato, Bernadete Liphaus, Maria Lígia Nerger, Monica Conde, Ana Freitas Ribeiro

**Affiliations:** 1 Secretaria de Estado da Saúde de São Paulo, Instituto Adolfo Lutz, Centro de Bacteriologia, Núcleo de Meningites, Pneumonias e Infecções Pneumocócicas, São Paulo, SP, Brasil; 2 Universidade Federal de Goiás, Instituto de Patologia Tropical e Saúde Pública, Goiânia, GO, Brasil; 3 Fundação Oswaldo Cruz, Instituto Oswaldo Cruz, Rio de Janeiro, RJ, Brasil; 4 Secretaria de Estado da Saúde de São Paulo, Centro de Vigilância Epidemiológica, Divisão de Doenças de Transmissão Respiratória, São Paulo, SP, Brasil; 5 Secretaria Municipal da Saúde, Coordenação de Vigilância em Saúde, Centro de Controle de Doenças, São Paulo, SP, Brasil; 6 Instituto de Infectologia Emílio Ribas, São Paulo, SP, Brasil

**Keywords:** Haemophilus influenzae, type b, nasopharyngeal carriage, Hib conjugate vaccine, Brazil

## Abstract

The aim of the present study was to assess the prevalence of* Haemophilus influenzae *type b (Hib) nasopharyngeal (NP) colonisation among healthy children where Hib vaccination using a 3p+0 dosing schedule has been routinely administered for 10 years with sustained coverage (> 90%). NP swabs were collected from 2,558 children who had received the Hib vaccine, of whom 1,379 were 12-< 24 months (m) old and 1,179 were 48-< 60 m old. Hi strains were identified by molecular methods. Hi carriage prevalence was 45.1% (1,153/2,558) and the prevalence in the 12-< 24 m and 48-< 60 m age groups were 37.5% (517/1,379) and 53.9% (636/1,179), respectively. Hib was identified in 0.6% (16/2,558) of all children in the study, being 0.8% (11/1,379) and 0.4% (5/1,179) among the 12-< 24 m and 48-< 60 m age groups, respectively. The nonencapsulate Hi colonisation was 43% (n = 1,099) and was significantly more frequent at 48-< 60 m of age (51.6%, n = 608) compared with that at 12-< 24 m of age (35.6%, n = 491). The overall resistance rates to ampicillin and chloramphenicol were 16.5% and 3.7%, respectively; the co-resistance was detected in 2.6%. Our findings showed that the Hib carrier rate in healthy children under five years was very low after 10 years of the introduction of the Hib vaccine.

The asymptomatic nasopharyngeal (NP) carriers of *Haemophilus influenzae*type b (Hib) play an important role in the dissemination of this microorganism in a population. Hib residing in the nasopharynx can invade the bloodstream or tissue of the host causing diseases (Moxon 1986). Worldwide Hib conjugate vaccine has proven to be highly effective in the prevention of invasive diseases caused by Hib. Vaccination against Hib also prevents the Hib colonisation in vaccinated children, consequently decreasing its transmission to unvaccinated population that may result in herd effect (Takala et al. 1991, Mohle-Boetani et al. 1993, Murphy et al. 1993, Jacups 2011).

Globally, countries have introduced the Hib vaccine using different schedules, including three primary doses (3p+0 dose), two primary doses plus a booster (2p+1 dose) or three primary doses plus a booster (3p+1 dose). To date, there is no clear evidence suggesting that any one schedule is likely to provide better protection against Hib invasive disease compared with the others (WHO 2010).

In July 1999, the Brazilian National Immunisation Program (NIP) introduced the monovalent Hib conjugate vaccine (PRP-Hib) into the universal infant immunisation program, using a primary series of three doses scheduled at two, four and six months of age without a booster dose (3p+0 dose). In 2002, the PRP-Hib vaccine was replaced by the combined DPT+Hib vaccine, which was in turn replaced by the pentavalent DPPT-Hib-HB in 2012; the vaccine schedule of 3p+0 dose has remained consistent to date. Since the introduction of the Hib vaccine in Brazil, the incidence of Hib meningitis declined sharply after 2001 coinciding with the consolidation of vaccination against Hib (MS/SVS/PNI 2010).

Surveillance of Hib carriage has been strongly recommended by the World Health Organization (WHO) as an important tool to investigate the frequency of vaccine type among the vaccinated population, as well as to evaluate the impact of vaccination (WHO 2010). In general, high rates of colonisation correlate with high rates of invasive disease (Garcia et al. 2012). In Brazil, studies investigating Hib NP carriage are scarce; only four studies have been published to date. Two were conducted before the introduction of the Hib vaccine in the NIP, in 1998, in the South and Southeast Regions of Brazil (Forleo-Neto et al. 1999, Bricks et al. 2004); the other two studies were carried out in the Southeast and in Central-West Regions and evaluated Hib carrier status after three and five years of Hib vaccination (Silva et al. 2006,Carvalho et al. 2011). This paucity of data prompted us to investigate the long term prevalence of Hib NP colonisation in a Brazilian population. The aim of the present study was to report the prevalence of Hib NP colonisation among healthy children living in the municipality of São Paulo, where Hib vaccination using a 3p+0 dose schedule has been routinely administered for 10 years. The antimicrobial susceptibility of Hi isolates was also investigated.

## SUBJECTS, MATERIALS AND METHODS

*Study population immunised with the Hib vaccine* - The present cross-sectional study was conducted in the municipality of São Paulo (population in 2010: 11,253,503 inhabitants) (IBGE 2010), the largest city in Brazil, during vaccination campaign in 2010. The enrollment of study participants was conducted at 16 primary health units (PHUs) conveniently selected due to high demand of children attended to these PHUs. Health children living in the urban area of São Paulo were recruited according to the spontaneous demand at PHUs. Two age groups were studied. Children aged 12-< 24 months (m) were selected because they had received the Hib vaccine a year before sample collection and children aged 48 up to < 60 m (48-< 60 m) were selected because they had been vaccinated in the five years before the study. Only one child per household was recruited.

*Sample size* - The number of study participants in each age group was calculated based on the 0.7% Hib carriage rate (error 0.5%) observed in previous studies conducted in vaccinated children in two Brazilian cities (Forleo-Neto et al. 1999, Carvalho et al. 2011). Thus, we estimated a target population of 1,100 subjects for each age group.

*Ethical considerations* - The study was approved by the Ethical Committee of the Public Health Faculty of University of São Paulo (protocol 1969). Before NP specimens were collected, parents or legal guardians of children received an appropriated explanation of the study and information on the risks and discomforts of the collection procedure. Children or parents/legal guardian that refused to participated in the study were not enrolled. Written informed consent was obtained from each parents or legal guardians of children.

*Data and specimen collection* - A questionnaire was administered by field-workers to parents/legal guardians of each participant to obtain demographic data. The Hib vaccine schedule (3p+0 dose) was ensured by checking the vaccine card at the time of NP specimen collection. The exclusion study criteria were refusal to participate, age not within the age range, not living in the city of São Paulo, incomplete Hib vaccination or received a booster dose (3p+1 dose) and used antibiotics or had fever (≥ 38.5ºC) within the seven days before sample collection. Specimen collection was performed in three different days of vaccination campaign in 2010: March 22 (n = 681), June 12 (n = 577) and August 14 (n = 1,300). NP specimens were obtained transnasally using a flexible, sterile swab with a flocked nylon tip (Copan, USA) by trained nurses according to WHO procedure (O’Brien et al. 2003). One NP sample was obtained per child. Swabs were immediately inoculated in 1 mL of skim milk-tryptone-glucose-glycerine (STGG) transport medium (O’Brien et al. 2001). Specimens were transported in a cold-box to the laboratory within 4-5 h of collection, where each tube was vortexed at high speed for 10-20 s and immediately frozen at -70ºC.

*Isolation and identification* - A 70 µL sample of the STGG transport medium from each specimen was inoculated on a chocolate agar plate supplemented with 300 µg bacitracin mL^-1^ for the selective isolation of Hi. The plates were incubated in 5% CO_2_ for 24-48 h. One colony showing the typical morphology of Hi, i.e., a smooth, grayish and wet colony was selected for DNA extraction and species identification using a real-time quantitative PCR (RT-PCR) assay targeting the protein D gene (*hpd 3*) as previously described (Wang et al. 2012). The capsular typing of confirmed Hi isolates was performed by conventional PCR for the presence of the*bexA *gene to detected encapsulated Hi and for all six capsule-specific genes (van Ketel et al. 1990, Falla et al. 1995). The standard strains used as positive controls in the RT-PCR and PCR assays were Hia ATCC9006, Hib ATCC35533, Hic ATCC9007, Hid ATCC9332, Hie ATCC8142 and Hif ATCC9833.

*Antimicrobial susceptibility testing* - A total of 272 (23.6%) Hi strains, including all encapsulated Hi (n = 54, 2.1%) and 20% (n = 218) of nonencapsulate Hi (NTHi) isolates randomly selected by using the Excel program (v.2010.11), were tested for antimicrobial susceptibility. Minimal inhibitory concentration (MIC) for ampicillin (AMP) and chloramphenicol (CHO) were determined by broth microdilution. Susceptibility criteria were defined according to the Clinical Laboratory Standards Institute guidelines (CLSI 2009). The β-lactamase activity was determined by chromogenic cephalosporin testing using nitrocefin as substrate and following the manufacturer’s instruction (Oxoid, EUA). Hi ATCC 49247 was used as a quality control strain for MICs; *Staphylococcus aureus* ATCC29213 and Hi ATCC 49247 were used as positive and negative controls, respectively, in the β-lactamase testing.

*Statistical analysis* - Analysis were performed using the SPSS v.18.0 (SPSS, Inc, USA) software package. Differences on Hi type and NTHi rates were evaluated by Fisher’s test or chi-squared test; a p-value < 0.05 was considered to be statistically significant.

## RESULTS

A total of 2,615 children were enrolled in the present study; 38 (1.4%) were excluded because they were not within the age ranges, 15 (0.6%) were from other municipality than São Paulo and four (0.1%) were excluded because sample collection had been performed in duplicate. Therefore, 2,558 children were included in the study, of whom 1,379 (53.9%) were children aged 12-< 24 m (median age = 18 m) and 1,179 (46.1%) were children aged 48-< 60 m (median age = 53 m).

The Table displays the prevalence of Hi carriage, Hi types and NTHi by age groups. On the whole, Hi colonisation rate was 45.1% (1,153/2,558), being significantly higher among children aged 48-< 60 m (53.9%, 636/1,179) compared with children aged 12-< 24 m (37.5%, 517/1,379).

**TABLE:** Prevalence of *Haemophilus influenzae* (Hi) nasopharyngeal carriage, Hi types and nonencapsulated Hi (NTHi) by age groups in healthy vaccinated children*a*, municipality of São Paulo, Brazil, 2010

Hi carrier status	Total (n = 2,558)	Age groups (months)		
		12-< 24 (n = 1,379)	48-< 60 (n = 1,179)	

	n (%)	n (%)	n (%)	p
Carrier	1,153 (45.1)	517 (37.5)	636 (53.9)	< 0.001

b	16 (0.6)	11 (0.8)	5 (0.4)	> 0.05
a	16 (0.6)	6 (0.4)	10 (0.8)	> 0.05
c	3 (0.1)	1 (0.1)	2 (0.2)	-
d	3 (0.1)	1 (0.1)	2 (0.2)	-
e	15 (0.6)	7 (0.5)	8 (0.7)	> 0.05
f	1 (0)	0 (0)	1 (0.1)	-
Encapsulate-Hi	54 (2.1)	26 (1.9)	28 (2.4)	> 0.05
NTHi	1,099 (43)	491 (35.6)	608 (51.6)	< 0.001
Non-Hi carrier	1,405 (54.9)	862 (62.5)	543 (46.1)	< 0.001

a : Hi type b conjugate vaccine.

Hib was found in 0.6% (n = 16) of 2,558 children, 0.8% (n = 11) and 0.4% (n = 5) in children aged 12-< 24 m and 48-< 60 m, respectively; Hi type a (Hia) was also detected in 0.6% (n = 16) of children and the other Hi types were found in low prevalence. The overall NP colonisation by encapsulate Hi was 2.1% (n = 54); among children aged 12-< 24 m and 48-< 60 m it was 1.9% (n = 26) and 2.4% (n = 28), respectively. The NTHi carriage rate was 43% (n = 1,099), being significantly more frequent in ages 48-< 60 m (51.6%, n = 608) compared with ages 12-< 24 m (35.6%, n = 491).

The overall rates of resistance to AMP and CHO were 16.5% (45/272) and 3.7% (10/272), respectively; co-resistance to AMP and CHO was detected in 2.6% (7/272) of isolates. Among encapsulate isolates, a resistance rate of 9.2% (5/54) was found for both antibiotics; NTHi isolates showed 18.3% (40/218) and 2.3% (5/218) resistance to AMP and CHO, respectively. All AMP-resistant isolates were β-lactamase producers.

## DISCUSSION

In the present survey, we observed that Hib was rarely identified as a NP coloniser (0.6%) in the vaccinated paediatric population in Brazil; in addition, there were no significant differences in the prevalence of Hib between the two age groups. Only 0.8% of children who had been vaccinated within six-12 m before sample collection (age group 12-< 24 m) were Hib carriers. Children belonging to the oldest age group (48-< 60 m), who had received the vaccine in the earlier five years before the present study, also had a low rate of Hib colonisation (0.4%). Data that corroborate with the low level of Hib dissemination in São Paulo is the low incidence rates of Hib meningitis (in 2010, 3.0/100,000 inhabitants of in children younger than one year and 0.65/100,000 in children between one-four years) (CVE 2014).

A low prevalence of Hib NP carriers was found in two studies previously conducted in Brazil involving vaccinated children attending day-care centres. One study that was performed three years after vaccination (2002-2003) among children 2-39 m of age living in the city of Ribeirão Preto, Southeast Brazil, found only 1% of Hib NP colonisation (Silva et al. 2006); another study performed six years after vaccination (2005) among children 2-59 m age in Goiânia, Central-West Region of the country, also reported a very low rate (0.7%) of Hib colonisation of the nasopharynx (Carvalho et al. 2011). Thus, despite temporal and regional differences among studies, as well as differences among study designs and laboratory methodologies, all have observed a low prevalence of Hib carriers, suggesting that the Hib vaccine schedule of 3p+0 dose incorporated with high vaccine coverage since 1999 has been effective in keeping the Hib circulation at a low level in this population. Importantly, we observed a low percentage of Hib colonisation among the oldest children, which indicates a long-term protection of Hib vaccination.

Similar to studies conducted in other countries, Hib colonisation rates found in these studies among unvaccinated children attending day-care centres were high, in the range of 4.8-7.3% (Forleo-Neto et al. 1999, Bricks et al. 2004). Since the widespread introduction of the Hib vaccine worldwide, several surveys have demonstrated a reduction in the prevalence of Hib colonisation over time, reporting low rates ranging from 1.5-< 0.1% (Mohle-Boetani et al. 1993, Barbour 1996, Millar et al. 2000, MacVernon et al. 2004) or even no isolation of Hib (Thomas et al. 2011, Lowther et al. 2012). On the other hand, persistence of Hib carriage in specific vaccinated children (e.g., Alaskan children) has been observed (Galil et al. 1999, Perdue et al. 2000, Singleton et al. 2000). Similar to studies conducted in other locations, we observed a high prevalence of NTHi colonisation, which was significantly higher among older children, while non-b encapsulate Hi was far less prevalent (Raymond et al. 2001, Barbosa-Cesnik et al. 2006, Sá-Leão et al. 2008, Carvalho et al. 2011, Puig et al. 2014). Interestingly, in the present study, the prevalence of carriers with Hib, Hia and Hie were similar, while Hic, Hid and Hif were rarely isolated. Globally, NTHi is an important cause of otitis media during childhood (Murphy et al. 2009), although it is less frequent as a cause of invasive disease. Nevertheless, after introduction of Hib vaccination, a relative increase in the number of cases of invasive disease caused by NTHi as well as encapsulate non-b Hi has been reported, mainly in populations with co-morbidities (Bajanca et al. 2004, Campos et al. 2004, Bruce et al. 2008, Adam et al. 2010, Giufrè et al. 2011, Ladhani et al. 2012). In Brazil, an increased number of NTHi as well as Hia and Hif as causes of meningitis was associated with the improvement of laboratorial surveillance in the post-Hib vaccine period, rather than dissemination of non-b Hi in the population (Zanella et al. 2011). Thus, these findings need to be monitored further and more extensively evaluated.

With regard to Hi antimicrobial resistance, we observed low resistance to AMP (16.5%) and CHO (3.6%). Comparable rates have been observed in the past several years (2010-2013) among invasive Hi isolates from Brazilian children up to five years of age (AMP: 18.3%; CHO: 6.3%) (Zanella et al. 2011, SIREVA II 2014). In spite of differences among study methodologies and settings, high rates of AMP resistance in health young children have been reported in Spain (24%) (Puig et al. 2014), India (22.9%) (Jain et al. 2005) and other countries (Rennie & Ibrahim 2005), which prompts us to be wary because the nasopharynx is the ecological niche for the acquisition of new Hi strains and exchange of resistance genes.

Our findings showed that the Hib carrier rate in healthy children under five years was very low after 10 years of the introduction of the Hib vaccine. Monitoring of Hib carriage is recommended to evaluate the transmission of Hib and should be coordinated with early intervention to effectively control the recurrence of invasive Hib disease.
